# Workout with a Smartwatch: A Cross-Sectional Study of the Effects of Smartwatch Attributes on Flow Experience and Exercise Intentions Depending on Exercise Involvement

**DOI:** 10.3390/healthcare11233074

**Published:** 2023-11-30

**Authors:** Jihyeon Oh, Daehwan Kim

**Affiliations:** Department of Marine Sports, Division of Smart Healthcare, Pukyong National University, Busan 48513, Republic of Korea; ohjihuny@snu.ac.kr

**Keywords:** healthcare, exercise, smartwatch attributes, flow experience, exercise involvement

## Abstract

Smartwatches are emerging as effective tools to promote exercise and physical activities in the healthcare industry. However, little is known about how smartwatch attributes facilitate exercise and for whom such attributes are more effective for exercise. Accordingly, the purpose of this study was to explore the structural relationship between smartwatch attributes, flow experience, and continued exercise intentions and to examine the moderating role of exercise involvement in the structural relationship. For this, a total of 600 participants were recruited via a professional survey firm in South Korea based on a multi-stage random sampling method and used for data analyses, including confirmatory factor analysis (CFA), structural equation modeling (SEM), and multi-group SEM. All survey items were adopted from the existing literature on healthcare, flow experience, and wearable device technologies. The results revealed that smartwatch attributes, including interactivity (γ = 0.234, *p* < 0.001/γ = 0.235, *p* < 0.001), autonomy (γ = 0.225, *p* < 0.001/γ = 0.172, *p* < 0.001), wearability (γ = 0.104, *p* < 0.05/γ = 0.106, *p* < 0.05), convenience (γ = 0.209, *p* < 0.001/γ = 0.214, *p* < 0.001), and experiential novelty (γ = 0.221, *p* < 0.001/γ = 0.281, *p* < 0.001) enhanced flow experience (absorption/enjoyment) during exercise. Furthermore, flow experience (absorption/enjoyment) was found to positively influence exercise intention (β = 0.511, *p* < 0.001/β = 0.239, *p* < 0.001). Lastly, exercise involvement was found to modulate the structural relationships among smartwatch attributes, flow experience, and exercise intention (∆χ^2^ = 23.231, ∆*df* = 12, *p* < 0.05). By investigating these dynamics, this study contributes to shared knowledge not only in the healthcare literature but also in the wearable-technology literature. The results of the current study also provide useful guidelines for practitioners in the wearable-device and healthcare industries to develop optimal features of smartwatches for exercise and physical activities.

## 1. Introduction

There is a plethora of evidence showing that exercise not only slows aging but also helps individuals lead physically and mentally healthier lives [1,2]. Numerous governments have promoted the importance of exercise because it significantly reduces healthcare costs and improves citizens’ quality of life [3]. Despite the importance of exercise and physical activities in healthcare, there are diverse barriers that deter individuals from engaging in them [4,5]. One of the main barriers is a lack of motivation for exercise and physical activities. To solve this issue, practitioners and scholars have focused on smartwatches, which are the first commercialized wearable device technology and the most widely diffused in modern society [6], to break down such barriers because they believe smartwatches can effectively motivate individuals to participate in exercise and physical activities. In this regard, it is important to understand what specific attributes of smartwatches encourage users to participate in physical activities and exercise and how such behavior is activated by smartwatch attributes.

However, little is known about the psychological mechanisms underlying the effects of smartwatch attributes on continued exercise intentions. Specifically, previous studies mainly focused on the effect of smartwatch attributes on smartwatch adoption by relying on the technology acceptance model (TAM) or its variations (e.g., extended technology acceptance model [ETAM]) [7,8,9,10,11,12,13,14]. Although these studies provide meaningful insight into how an individual initially adopts wearable-device technologies, it is also imperative to understand what smartwatch attributes help individuals engage in exercise and physical activities when considering such devices’ main usage (i.e., healthcare) [15,16]. Furthermore, the existing literature on the relationship between smartwatch attributes and user behaviors has tended to focus on a certain target group [10,16,17,18] or consider users as a homogenous group rather than heterogeneous groups in which individuals have diverse characteristics [11,13,19,20]. As Venkatesh et al. [21] state, different contexts inspire new insight into existing theories, so considering user characteristics is essential for an in-depth understanding of the relationship between smartwatch attributes and user behaviors.

To fill the gaps in the existing literature on the role of smartwatches in healthcare, the present study aimed to investigate how smartwatch attributes help individuals engage in exercise by relying on the theory of flow experience [22,23,24], because the concept of flow experience is known as a key predictor of participation in diverse physical activities [25,26,27,28]. Moreover, the current study focused on the concept of exercise involvement because not only is it the most relevant criterion to segment smartwatch users for their exercise but it could also change users’ information processing and the evaluative criteria for smartwatches depending on its level (high or low). Overall, the purpose of this study was to explore the structural relationship among smartwatch attributes, flow experience, and continued exercise intentions and to examine the moderating role of exercise involvement in the structural relationship.

For these objectives, the present study reconceptualized smartwatch attributes because it focused on what features help users engage in exercise and physical activities, so some features irrelevant to the research context (e.g., price, brand, design aesthetics) were excluded. Therefore, the current study selected five main attributes: interactivity, perceived autonomy, wearability, perceived convenience, and experiential novelty, based on the existing literature on smartwatch attributes [6,19,29,30,31,32]. First, interactivity refers to the extent to which a smartwatch enables the user to communicate with other users in the mediated environment [19]. Second, the concept of perceived autonomy denotes the ability of smartwatches to conduct a certain task with a specific goal in an automatic and independent manner [19,33,34]. Third, the wearability of smartwatches has been largely ignored by academia, but it is a key determinant of exercise experience with smartwatches [35]. Fourth, another main benefit of wearing a smartwatch compared to traditional wristwatches is its technological convenience, which allows users to easily and ubiquitously deal with their daily work [32]. Lastly, the present study adopted the concept of experiential novelty as a key attribute in the context of exercise with smartwatches and defined it as the degree to which a smartwatch offers new and unique experiences when users participate in exercise with the smartwatch. Taken together, the present study assumed that all these attributes enhanced flow experience, which is a psychological state involving cognitive absorption and enjoyment, based on the existing literature [36,37,38,39,40].

The importance of flow experience has been highlighted in a wide range of contexts because it is strongly related to positive outcomes of exercise, including exercise satisfaction and intrinsic rewards, performance enhancement, health-related quality of life, perceived health improvement, mental health, well-being, and adherence to physical activities and exercise, among others [25,26,27,28,41,42,43,44]. Among these benefits of flow experience, in particular, the current study focused on the relationship between flow experience and intention to continuously engage in exercise using smartwatches due to its theoretical and practical implications in the field of healthcare. Flow theory suggests that a flow state makes exercise an autotelic experience generating intrinsic rewards, which can be an important foundation for adherence to exercise [25].

**Figure 1:**
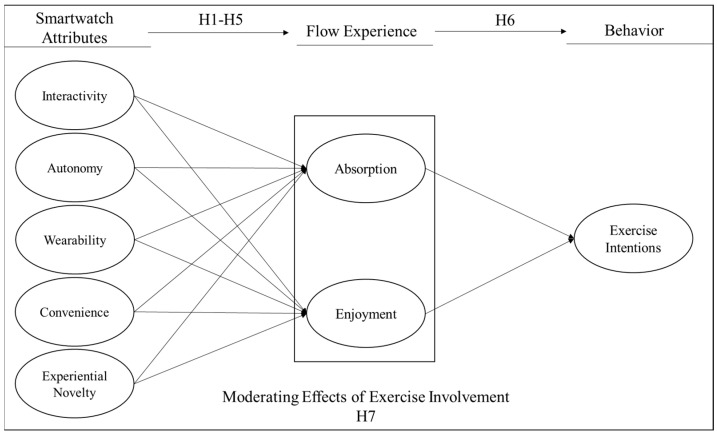
Hypothesized research model.

Meanwhile, there are several theoretical backgrounds offering a fundamental logic behind the different patterns of the structural relationship among smartwatch attributes, flow experience, and exercise intention, including the elaboration likelihood model (central vs. peripheral routes) [45] and the regulatory focus theory (promotion vs. prevention focus) [46]. These two theoretical perspectives similarly posit that individuals tend to focus on different attributes of a certain object depending on their level of involvement in the object or activity. Specifically, individuals with high involvement are likely to focus on the utilitarian and central components of the object or activity, whereas individuals with low involvement are likely to focus on hedonic and peripheral components. Applying this logic to the present study, we assumed that interactivity and experiential novelty of smartwatch attributes as well as enjoyment of flow experience would be more important to individuals who were less involved in exercise. In contrast, autonomy, wearability, and convenience of smartwatch attributes as well as absorption of flow experience would be more important to individuals who were highly involved in exercise. All in all, the following hypotheses were developed and are visualized in Figure 1:

**Hypothesis** **1.** *The interactivity of smartwatches positively influences flow experience (absorption and enjoyment) during exercise*.

**Hypothesis** **2.** *The autonomy of smartwatches positively influences flow experience (absorption and enjoyment) during exercise*.

**Hypothesis** **3.** *The wearability of smartwatches positively influences flow experience (absorption and enjoyment) during exercise*.

**Hypothesis** **4.** *The convenience of smartwatches positively influences flow experience (absorption and enjoyment) during exercise*.

**Hypothesis** **5.** *The experiential novelty of smartwatches positively influences flow experience (absorption and enjoyment) during exercise*.

**Hypothesis** **6.** *Flow experience (absorption and enjoyment) positively influences exercise intention using smartwatches*.

**Hypothesis** **7.** *Exercise involvement moderates the structural relationship between smartwatch attributes, flow experience, and exercise intention*.

## 2. Methods

### 2.1. Participants and Procedure

The present study collected data based on a cross-sectional design targeting individuals who use smartwatches for their exercise in South Korea as the research population. Specifically, the research participants were randomly selected utilizing a professional online survey institute (Embrain: https://embrain.com/, accessed on 30 August 2023) which currently has 1,708,434 panels across all provinces in South Korea. For the representativeness of the sample, the institute used a multi-stage random sampling method based on demographic characteristics to choose research participants. Additionally, sample cases in incomplete responses or recurring patterns were identified and eliminated via the rigorous survey system. To ensure the appropriateness of the sample, the survey included two screening questions: (a) “Do you have a smartwatch?” and (b) “Have you ever used a smartwatch for your exercise or physical activities?” Participants had to say “yes” to participate in the main survey. As a result, a total of 600 individuals were obtained and used in the data analysis. The obtained data consisted of an equal proportion of gender. The average age of the participants was 40.05 years (SD = 10.73). The majority of participants used Samsung smartwatches (n = 342, 57.0%), followed by Apple smartwatches (n = 183, 30.5%), etc. (n = 75, 12.5%). Additionally, 61.5% of the participants (n = 369) exercised 2–4 times a week, followed by 19.7% (n = 118) with 5–7 times, and 18.8% (n = 113) only once a week.

### 2.2. Instrument

Nine constructs were measured using existing scales on 7-point Likert scales (1 = “not at all”, 7 = “very much”). Specifically, the measurement items for smartwatch attributes including interactivity (3 items), autonomy (4 items), wearability (3 items), convenience (3 items), and experiential novelty (4 items) were adopted and modified from the existing literature on wearable devices and user experiences [47,48,49]. Flow experience was measured using three items for absorption and three items for enjoyment that were adopted and modified from Kim and Ko’s study [39]. Exercise intention was measured using three items adopted and modified from Stanley et al. [50]. Exercise involvement, which was used as a grouping variable (high vs. low), was measured using three items adapted from Zaichkowsky’s research [51]. Taken together, 29 items were included in the theorized measurement model. Lastly, the measurement items were translated into Korean by a bilingual author and then back-translated into an English version. We compared the translated version with the original version and resolved several minor differences via discussion and agreement to finalize the measurement items.

### 2.3. Data Analysis

Before testing the research hypotheses, descriptive statistics were analyzed to investigate participants’ characteristics and to identify potential outliers. Then, a confirmatory factor analysis (CFA) was conducted to examine the validity and reliability of the measurement model using Mplus v. 8.4. Furthermore, a series of measurement invariance tests between the high-exercise-involvement group and low-exercise-involvement group was performed to ensure that participants in those two groups equally interpreted the measurement items. For hypothesis testing, we conducted structural equation modeling (SEM) and multigroup SEM using Mplus v. 8.4.

## 3. Results

### 3.1. Measurement Model Validation

First of all, a confirmatory factor analysis was performed to evaluate the validity and reliability of the measurement model based on several fit indices suggested by Hair et al. [52]. The results indicated an acceptable fit between the measurement model and the data (χ^2^/*df* = 854.792/341 = 2.507, CFI = 0.969, TLI = 0.963, RMSEA = 0.050, SRMR = 0.046). All factor loadings of the measurement items were found to be statistically significant and above 0.70 (Table 1). The average variance extracted (AVE) values ranged from 0.614 (autonomy) to 0.864 (enjoyment), and the composite reliability (CR) coefficients ranged from 0.856 (interactivity) to 0.950 (enjoyment). These results ensured convergent validity and reliability of the measurement model [53,54]. Furthermore, the correlation coefficients between the constructs ranged from 0.185 (exercise involvement and wearability) to 0.889 (absorption and enjoyment), and the square of these correlation coefficients was lower than AVE values (Table 2). This result is evidence for the discriminant validity of the measurement model [53,54]. All in all, we concluded that construct validity and reliability of the measurement model were established.

### 3.2. Measurement Model Invariance Test

Before hypothesis testing, measurement invariance between the two groups (high exercise involvement vs. low exercise involvement) was assessed based on configural and metric invariance in the hierarchical models [54]. First of all, the current study developed Model 1 in which the number of constructs and the pattern of factor loadings were equally set but all parameters were freely estimated in each of the groups in order to test configural invariance. Model 1 should indicate an acceptable model fit to ensure configural invariance [54]. The results of the configural invariance test showed an acceptable fit (χ^2^/df = 1207.370/542 = 2.228, CFI = 0.953, TLI = 0.943, RMSEA = 0.064, SRMR = 0.055), and thus it was concluded that configural invariance of the measurement model was established. Second, we developed Model 2 in which all factor loadings were constrained to be the same across the groups to test metric invariance. The results showed that there was no statistically significant difference between Model 1 and Model 2 (∆χ2 = 16.446, ∆df = 18, *p* = 0.562). Therefore, metric invariance between high-exercise-involvement group and low-exercise-involvement group was ensured (Table 3) [54].

### 3.3. Hypothesis Testing

To test the established hypotheses in this study, structural equation modeling (SEM) was conducted. The results showed that the theorized research model had an acceptable fit between the structural model and the data (χ^2^/df = 1239.890/277 = 4.476, CFI = 0.935, TLI = 0.923, RMSEA = 0.076, SRMR = 0.081). The results also revealed that smartwatch attributes, including interactivity (H1: standardized γ = 0.234, *p* < 0.001), autonomy (H2: standardized γ = 0.225, *p* < 0.001), wearability (H3: standardized γ = 0.104, *p* < 0.05), convenience (H4: standardized γ = 0.209, *p* < 0.001), and experiential novelty (H5: standardized γ = 0.221, *p* < 0.001) had a positive impact on the absorption of flow experience. Additionally, interactivity (H1: standardized γ = 0.235, *p* < 0.001), autonomy (H2: standardized γ = 0.172, *p* < 0.001), wearability (H3: standardized γ = 0.106, *p* < 0.05), convenience (H4: standardized γ = 0.214, *p* < 0.001), and experiential novelty (H5: standardized γ = 0.281, *p* < 0.001) positively affected enjoyment of flow experience. Therefore, hypothesis 1 to hypothesis 5 were all supported. Regarding the effect of flow experience on exercise intention, the results indicated that both absorption (H6: standardized β = 0.511, *p* < 0.001) and enjoyment (H6: standardized β = 0.239, *p* < 0.001) had a positive impact on exercise intention. Accordingly, hypothesis 6 was tenable.

Meanwhile, multi-group SEM was performed to test the moderating effect of exercise involvement on the structural model (H7). For this analysis, the participants were divided into two groups (high vs. low level of exercise involvement) using the mean score of exercise involvement as a criterion. After that, we developed Model 3 in which all path coefficients were freely estimated, and Model 4 in which all path coefficients were constrained to be the same across the two groups. The results of the chi-square difference test revealed that there was a statistically significant difference between Model 3 and Model 4 (∆χ^2^ = 23.231, ∆*df* = 12, *p* < 0.05), suggesting a moderating effect of exercise involvement on the structural model (Table 3). Therefore, hypothesis 7 was accepted.

**Figure 2:**
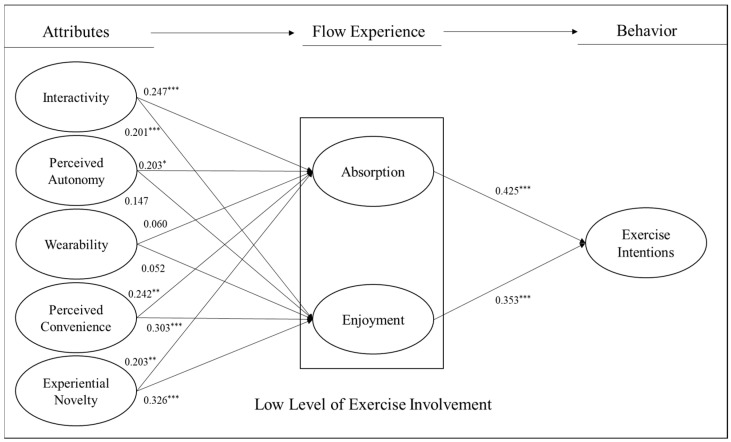
Path Coefficients in the group of low level of exercise involvement. Note: * *p* < 0.05, ** *p* < 0.01, *** *p*< 0.001.

Specifically, interactivity (γ = 0.247, *p* < 0.001), autonomy (γ = 0.203, *p* < 0.05), convenience (γ = 0.242, *p* < 0.01), and experiential novelty (γ = 0.203, *p* < 0.01) had a positive impact on the absorption of flow experience, but wearability had no significant effect on it (γ = 0.060, *p* = 0.488) in the low-exercise-involvement group (Figure 2). Furthermore, interactivity (γ = 0.201, *p* < 0.001), convenience (γ = 0.303, *p* < 0.001), and experiential novelty (γ = 0.326, *p* < 0.001) had a positive effect on the enjoyment of flow experience, but autonomy (γ = 0.147, *p* = 0.093) and wearability (γ = 0.052, *p* = 0.551) had no significant effect on it (γ = 0.060, *p* = 0.488). Concerning the effect of flow experience on exercise intention, the results indicated that both absorption (β = 0.425, *p* < 0.001) and enjoyment (β = 0.353, *p* < 0.001) had a positive impact on exercise intention.

**Figure 3:**
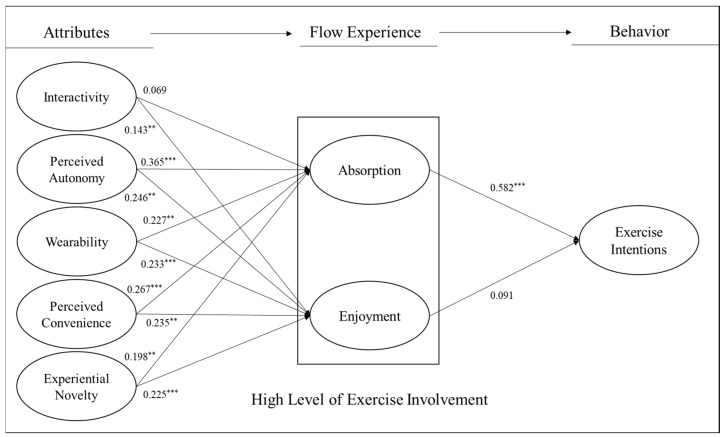
Path coefficients in the group of high level of exercise involvement. Note: ** *p* < 0.01, *** *p*< 0.001.

In the high-exercise-involvement group (Figure 3), autonomy (γ = 0.365, *p* < 0.001), wearability (γ = 0.227, *p* < 0.01), convenience (γ = 0.267, *p* < 0.001), and experiential novelty (γ = 0.198, *p* < 0.01) had a positive impact on the absorption of flow experience, but interactivity had no significant effect on it (γ = 0.069, *p* = 0.142). Additionally, all attributes (interactivity: γ = 0.143, *p* < 0.01; autonomy: γ = 0.246, *p* < 0.01; wearability: γ = 0.233, *p* < 0.01; convenience: γ = 0.235, *p* < 0.01; and experiential novelty: γ = 0.225, *p* < 0.001) had a positive effect on the enjoyment of flow experience. Interestingly, flow experience was found to partially influence exercise intention (absorption: β = 0.582, *p* < 0.001 and enjoyment: β = 0.091, *p* = 0.256).

## 4. Discussion

### 4.1. Interpretations of Results

Drawing on the literature on wearable technology, exercise, flow-experience theories, and dual-process models (e.g., the elaboration likelihood model and regulatory focus theory), the present study explored what attributes of smartwatches played a pivotal role in user experiences during exercise and how they influenced exercise intentions depending on the level of exercise involvement. The results of the hypothesis testing revealed that smartwatch attributes significantly enhanced flow experience, including absorption and enjoyment during exercise (H1–H5). These results are consistent with the findings in the literature on wearable technology and user experiences [19,29,30,32,33,34,35,55,56,57,58,59]. Additionally, these findings support the PAT model [40] in that an artifact plays a substantial role in user experiences (i.e., flow experience). In particular, the interactivity of smartwatches was found to most strongly enhance absorption and enjoyment during exercise. This finding implies that interactivity is the most important attribute of smartwatches when users participate in exercise. This interpretation is also supported by the literature on motivations for exercise that has suggested that social interaction with others is the main motivation for participation in exercise and physical activities [60,61,62]. Furthermore, as numerous studies have suggested that flow experience is a barometer and enhancer of the quality of a target task and thus is a powerful predictor of adherence to the task [25,26,27,28,39,41,42,43,44,63,64], our study also found that both absorption and enjoyment (flow experience) positively affected exercise intention with smartwatches (H6). In other words, the more flow state an individual experiences during exercise with a smartwatch, the more likely he or she is to participate in and adhere to exercise and physical activities with the smartwatch.

Meanwhile, exercise involvement was found to modulate the relationship between the variables in the research model (H 7). This finding means that the effects of smartwatch attributes on flow experience and the impact of flow experience on exercise intention vary depending the level of exercise involvement (low vs. high). Specifically, for the low-level exercise-involvement group, wearability had no impact on either absorption or enjoyment, both of which positively influenced exercise intention with smartwatches. For the high-level exercise-involvement group, only interactivity did not influence absorption, which solely had a positive impact on exercise intention. These findings imply that individuals who are less involved in exercise do not consider the wearability of smartwatches as an important attribute for their exercise, while individuals who are highly involved in exercise do not consider the interactivity as an important attribute for their exercise with smartwatches. Furthermore, absorption during exercise was an important aspect of exercise experience (i.e., flow experience) for both low- and high-level exercise-involvement groups, whereas enjoyment was not important for the high-level group. These results support the basic assumption of the dual-process theories, including the elaboration likelihood model and regulatory focus theory [45,46]. According to these theories, individuals with high exercise involvement should prioritize utilitarian and central components of smartwatches and exercise, whereas those with low exercise involvement should focus relatively more on hedonic and peripheral components of smartwatches and exercise. Indeed, in our study, individuals who were less involved in exercise tended to focus more on the hedonic aspects of exercise with smartwatches (interactivity, experiential novelty, and enjoyment) whereas those with high exercise involvement tended to prioritize utilitarian features of smartwatches and exercise (autonomy, wearability, and absorption).

### 4.2. Theoretical and Practical Implications

The findings of this study have several meaningful theoretical implications for the literature on wearable technology and healthcare. First, the present study extends the literature by elucidating what attributes of wearable technologies (e.g., smartwatches) affect user experiences during exercise and explicates how they ultimately encourage individuals to participate in exercise and physical activities. Specifically, the existing literature has mainly focused on what attributes of wearable devices affect wearable-device adoption based on the technology-acceptance models, so little is known regarding how such wearable devices help individuals engage in exercise and physical activities for their well-being. In this regard, the current study offers evidence regarding the role of wearable-device attributes in exercise experience and healthcare behavior (i.e., adherence to exercise). In particular, the present study offers evidence regarding what attribute (i.e., interactivity) of wearable devices is most important for users to experience an optimal state (i.e., flow experience) during exercise and how wearable devices encourage individuals to participate in exercise by illuminating the role of flow experience in exercise intention.

Moreover, the current study contributes to the literature on wearable-device technology and healthcare by revealing that dynamics within the research model, including smartwatch attributes, flow experience, and exercise intention varying depending on the level of exercise involvement. Previous studies tended to examine relationships among wearable device attributes, user experiences, and behaviors by only focusing on a certain target group [10,16,17,18] or considering users as a homogeneous group [11,13,19,20]. To fill this gap in the literature, the present study examined the moderating role of exercise involvement, which is a key variable in segmenting users of wearable devices for exercise, in the relationships among smartwatch attributes, flow experiences, and exercise intention. Therefore, this study offers meaningful insight into boundary conditions for the effect of smartwatch attributes on user experiences and exercise intentions.

Lastly, the present study also provides several practical implications to the smartwatch and healthcare industries. The benefits of smartwatch attributes in user experiences and adherence to exercise imply that a smartwatch can be a powerful medium for motivating individuals to participate in exercise and physical activity. In particular, the findings suggest that smartwatches (or applications for smartwatches) should be designed to maximize social-exchange experiences (i.e., interactivity) during exercise because such experiences are likely to amplify flow experience. Furthermore, the moderating effects of exercise involvement on the relationship among smartwatch attributes, flow experience, and exercise intention offer useful insight into the segmentation, targeting, and positioning (STP) strategy. Specifically, the findings suggest that utilitarian attributes of smartwatches (e.g., autonomy, wearability, and absorption) could appeal more to individuals who are highly involved in exercise, whereas hedonic attributes of smartwatches (e.g., interactivity, experiential novelty, and enjoyment) could appeal more to individuals who are less involved in exercise.

### 4.3. Limitations and Future Research Agendas

Despite the theoretical and practical contributions of the current study, it is necessary to discuss several limitations to provide insight for future research avenues. First, the present study focused on the concept of flow experience as a key indicator of the quality of exercise experiences because it seemed to capture both cognitive (absorption) and affective (enjoyment) aspects of exercise experiences. However, smartwatch attributes may lead to diverse and complex experiential aspects (e.g., self-control, social image, and utilitarian, hedonic, eudemonic, and social-needs fulfillment). Therefore, future research needs to explore theoretically and practically meaningful mediators between smartwatch attributes and exercise intentions. Second, although the present study focused on how smartwatch attributes facilitate exercise intentions, other technological aspects of smartwatches may help individuals engage in exercise and physical activities. For example, the concept of gamification [49] has been widely used to explain the relationship between media technology and user experiences. Therefore, testing a comprehensive model in which smartwatch attributes and gamification collaborate and influence user experiences during exercise may provide useful implications for the wearable technology and healthcare literature. Third, the present study considered exercise involvement as a user characteristic for segmentation. Thus, exploring the moderating effect of other user characteristics (e.g., gender, age, user innovativeness) may offer a deeper understanding of how smartwatch attributes influence user experiences and exercise intentions. Lastly, the present study collected data in South Korea, targeting individuals who had exercise experience with smartwatches based on a cross-sectional design. Accordingly, the results should be cautiously interpreted when generalizing them to other contexts. In this respect, future studies could explore how cultural differences affect the structural relationship between smartwatch attributes, flow experience, and exercise intention by setting cultural backgrounds as a moderating variable.

## 5. Conclusions

In conclusion, smartwatches are an effective tool in motivating individuals to engage in exercise by enhancing the quality of exercise experiences (i.e., flow experience). Specifically, it can be concluded that the main attributes of smartwatches, including interactivity, autonomy, wearability, convenience, and experiential novelty substantially enhance flow experience (absorption and enjoyment) during exercise, and such optimal experience leads individuals to adhere to exercise with smartwatches. Furthermore, we concluded that the impact of smartwatch attributes on flow experience and the effect of flow experience on exercise intention vary depending on the level of exercise involvement. By investigating these dynamics, this study contributes to the shared knowledge not only in the healthcare literature but also in the wearable technology literature. The results of the current study also provide useful guidelines for practitioners in the wearable-device and healthcare industries to develop optimal features of smartwatches for exercise and physical activities.

## Figures and Tables

**Table 1:** Summary results for confirmatory factor analysis (CFA).

Factors and Items	λ	C.R.	AVE
Interactivity		0.856	0.666
When I work out with my smartwatch, I can compare my amount of exercise with other users.	0.827		
When I work out with my smartwatch, I can connect to and communicate with other users.	0.759		
When I work out with my smartwatch, I can compare my exercise performance with other users on the app installed on my smartwatch.	0.859		
Autonomy		0.864	0.614
My smartwatch can automatically record my physiological information in anytime and everywhere.	0.766		
My smartwatch can automatically measure my physiological state and exercise performance.	0.753		
My smartwatch enables me access to the information about my physiological state and exercise performance in anytime and everywhere.	0.806		
My smartwatch performs tasks with my least effort and intervention	0.807		
Wearability		0.866	0.683
When I wear my smartwatch, I feel comfortable.	0.742		
I do not feel my smartwatch interferes my movements.	0.851		
When I wear my smartwatch, I do not feel any inconvenience	0.880		
Convenience		0.929	0.813
My smartwatch allows me to simultaneously perform many tasks during exercise.	0.868		
My smartwatch allows me to easily perform many tasks during exercise.	0.931		
My smartwatch allows me to conveniently perform many tasks during exercise.	0.905		
Experiential Novelty		0.948	0.819
Working out with the smartwatch gives me a unique experience.	0.807		
Working out with the smartwatch gives me a novel experience.	0.918		
Working out with the smartwatch gives me an unusual experience.	0.960		
Working out with the smartwatch gives me a new experience.	0.928		
Absorption		0.949	0.860
When I work out with my smartwatch, I am totally focused on it.	0.904		
When I work out with my smartwatch, I am totally engrossed in it.	0.950		
When I work out with my smartwatch, I am absorbed intensely.	0.929		
Enjoyment		0.950	0.864
Working out with the smartwatch is enjoyable.	0.925		
Working out with the smartwatch is exciting.	0.931		
Working out with the smartwatch is fun.	0.932		
Exercise Intention		0.933	0.824
I would like to continue working out with my smartwatch.	0.961		
It is highly likely for me to work out with my smartwatch.	0.945		
I will continue to work out with my smartwatch.	0.810		
Exercise Involvement		0.929	0.815
I am highly interested in exercise.	0.876		
Exercise is important to me.	0.905		
Exercise is of high value to me.	0.926		

**Table 2:** Correlations between constructs.

	1	2	3	4	5	6	7	8	9
1 Interactivity	**0.666**	0.100 *	0.073 *	0.105 *	0.084 *	0.190 *	0.194 *	0.141 *	0.092 *
2 Autonomy	*0.316*	**0.614**	0.303 *	0.318 *	0.157 *	0.285 *	0.256 *	0.317 *	0.093 *
3 Wearability	*0.271*	*0.535*	**0.683**	0.323 *	0.190 *	0.235 *	0.241 *	0.327 *	0.034 *
4 Convenience	*0.324*	*0.564*	*0.568*	**0.813**	0.328 *	0.342 *	0.355 *	0.247 *	0.066 *
5 Novelty	*0.289*	*0.396*	*0.436*	*0.573*	**0.819**	0.289 *	0.335 *	0.161 *	0.100 *
6 Absorption	*0.436*	*0.534*	*0.485*	*0.585*	*0.538*	**0.860**	0.790 *	0.465 *	0.230 *
7 Enjoyment	*0.441*	*0.506*	*0.491*	*0.596*	*0.579*	*0.889*	**0.864**	0.416 *	0.184 *
8 Intention	*0.375*	*0.563*	*0.572*	*0.497*	*0.401*	*0.682*	*0.645*	**0.824**	0.108 *
9 Involvement	*0.303*	*0.305*	*0.185*	*0.257*	*0.316*	*0.480*	*0.429*	*0.329*	**0.815**

Note: values * = squared values of the correlations between the constructs; bold = AVE values; italicized = correlations between the constructs.

**Table 3:** Results of model comparisons.

Model	Model Fit Indices	Model Comparison
Model 1: Measurement model without constraints	χ^2^/df = 1207.370/542, CFI = 0.953, TLI = 0.943, RMSEA = 0.064, SRMR = 0.055	
Model 2: Model 1 + Equal Factor Loadings	χ^2^/df = 1223.816/560, CFI = 0.953, TLI = 0.945, RMSEA = 0.063, SRMR = 0.058	∆χ2 = 16.446, ∆df = 18, *p* = 0.562
Model 3: Structural model without constraints	χ^2^/df = 1726.711/590, CFI = 0.919, TLI = 0.911, RMSEA = 0.080, SRMR = 0.089	
Model 4: Model 3 + Equal path coefficients	χ^2^/df = 1749.942/602, CFI = 0.918, TLI = 0.912, RMSEA = 0.080, SRMR = 0.092	∆χ^2^ = 23.231, ∆df = 12, *p* < 0.05

## Data Availability

The data used for the current study are available from the corresponding author upon reasonable request.

## References

[1] Sallis R.E. (2009). Exercise Is Medicine and Physicians Need to Prescribe It!. Br. J. Sports Med..

[2] Sari N. (2011). Exercise, Physical Activity and Healthcare Utilization: A Review of Literature for Older Adults. Maturitas.

[3] Sari N. (2009). Physical Inactivity and Its Impact on Healthcare Utilization. Health Econ..

[4] Tappe M.K., Duda J.L., Ehrnwald P.M. (1989). Perceived Barriers to Exercise among Adolescents. J. Sch. Health.

[5] Ebben W., Brudzynski L. (2008). Motivations and barriers to exercise among college students. J. Exerc. Physiol. Online.

[6] Jung Y., Kim S., Choi B. (2016). Consumer Valuation of the Wearables: The Case of Smartwatches. Comput. Human. Behav..

[7] Wu L.-H., Wu L.-C., Chang S.-C. (2016). Exploring Consumers’ Intention to Accept Smartwatch. Comput. Human. Behav..

[8] Dutot V., Bhatiasevi V., Bellallahom N. (2019). Applying the Technology Acceptance Model in a Three-Countries Study of Smartwatch Adoption. J. High Technol. Manag. Res..

[9] Kim K.J., Shin D.-H. (2015). An Acceptance Model for Smart Watches: Implications for the Adoption of Future Wearable Technology. Internet Res..

[10] Chen J., Wang T., Fang Z., Wang H. (2023). Research on Elderly Users’ Intentions to Accept Wearable Devices Based on the Improved UTAUT Model. Front. Public Health.

[11] Huarng K.-H., Yu T.H.-K., Fang Lee C. (2022). Adoption Model of Healthcare Wearable Devices. Technol. Forecast. Soc. Chang..

[12] Yang Q., Al Mamun A., Hayat N., Jingzu G., Hoque M.E., Salameh A.A. (2022). Modeling the Intention and Adoption of Wearable Fitness Devices: A Study Using SEM-PLS Analysis. Front. Public Health.

[13] Kim K.J. (2016). Round or Square? How Screen Shape Affects Utilitarian and Hedonic Motivations for Smartwatch Adoption. Cyberpsychology Behav. Soc. Netw..

[14] Larnyo E., Dai B., Larnyo A., Nutakor J.A., Ampon-Wireko S., Nkrumah E.N.K., Appiah R. (2022). Impact of Actual Use Behavior of Healthcare Wearable Devices on Quality of Life: A Cross-Sectional Survey of People with Dementia and Their Caregivers in Ghana. Healthcare.

[15] Daniel Ruby Smartwatch Statistics 2023: How Many People Use Smartwatches?. https://www.demandsage.com/smartwatch-statistics/.

[16] Moye R., Towns T., Skipper A., Rose D. (2022). Are Smartwatches Actually Used for Exercise? Evidence from HBCU Students. Am. J. Health Educ..

[17] Magni D., Scuotto V., Pezzi A., Del Giudice M. (2021). Employees’ Acceptance of Wearable Devices: Towards a Predictive Model. Technol. Forecast. Soc. Change.

[18] Farivar S., Abouzahra M., Ghasemaghaei M. (2020). Wearable Device Adoption among Older Adults: A Mixed-Methods Study. Int. J. Inf. Manag..

[19] Basha N.K., Aw E.C.-X., Chuah S.H.-W. (2022). Are We so over Smartwatches? Or Can Technology, Fashion, and Psychographic Attributes Sustain Smartwatch Usage?. Technol. Soc..

[20] Bölen M.C. (2020). Exploring the Determinants of Users’ Continuance Intention in Smartwatches. Technol. Soc..

[21] Venkatesh V., Thong J.Y.L., Xu X. (2012). Consumer Acceptance and Use of Information Technology: Extending the Unified Theory of Acceptance and Use of Technology. MIS Q..

[22] Csikszentmihalyi M. (1990). Flow: The Psychology of Optimal Experience.

[23] Csikszentmihalyi M. (1997). Finding Flow: The Psychology of Engagement with Everyday Life.

[24] Jackson S.A., Csikszentmihalyi M. (1999). Flow in Sports.

[25] Petosa R.L., Holtz B. (2013). Flow for Exercise Adherence: Testing an Intrinsic Model of Health Behavior. Am. J. Health Educ..

[26] Swann C., Jackman P.C., Schweickle M.J., Vella S.A. (2019). Optimal Experiences in Exercise: A Qualitative Investigation of Flow and Clutch States. Psychol. Sport Exerc..

[27] Schüler J., Brunner S. (2009). The Rewarding Effect of Flow Experience on Performance in a Marathon Race. Psychol. Sport Exerc..

[28] Cheng T.-M., Lu C.-C. (2015). The Causal Relationships among Recreational Involvement, Flow Experience, and Well-Being for Surfing Activities. Asia Pac. J. Tour. Res..

[29] Hsiao K.-L., Chen C.-C. (2018). What Drives Smartwatch Purchase Intention? Perspectives from Hardware, Software, Design, and Value. Telemat. Inform..

[30] Adapa A., Nah F.F.-H., Hall R.H., Siau K., Smith S.N. (2018). Factors Influencing the Adoption of Smart Wearable Devices. Int. J. Hum. Comput. Interact..

[31] Gopinath K., Sai L.P. (2023). A Study on the Positioning of the Brand Variants by Smartwatch Manufacturers: A Technometrics Approach. Technol. Anal. Strateg. Manag..

[32] Ogbanufe O., Gerhart N. (2018). Watch It! Factors Driving Continued Feature Use of the Smartwatch. Int. J. Hum. Comput. Interact..

[33] Cho W.-C., Lee K.Y., Yang S.-B. (2019). What Makes You Feel Attached to Smartwatches? The Stimulus–Organism–Response (S–O–R) Perspectives. Inf. Technol. People.

[34] Beer J.M., Fisk A.D., Rogers W.A. (2014). Toward a Framework for Levels of Robot Autonomy in Human-Robot Interaction. J. Hum. Robot. Interact..

[35] Jeong H., Kim H., Kim R., Lee U., Jeong Y. (2017). Smartwatch Wearing Behavior Analysis: A Longitudinal Study. Proc. ACM Interact. Mob. Wearable Ubiquitous Technol..

[36] Huang M.-H. (2003). Designing Website Attributes to Induce Experiential Encounters. Comput. Human. Behav..

[37] Skadberg Y.X., Kimmel J.R. (2004). Visitors’ Flow Experience While Browsing a Web Site: Its Measurement, Contributing Factors and Consequences. Comput. Human. Behav..

[38] Van Noort G., Voorveld H.A.M., Van Reijmersdal E.A. (2012). Interactivity in Brand Web Sites: Cognitive, Affective, and Behavioral Responses Explained by Consumers’ Online Flow Experience. J. Interact. Mark..

[39] Kim D., Ko Y.J. (2019). The Impact of Virtual Reality (VR) Technology on Sport Spectators’ Flow Experience and Satisfaction. Comput. Human Behav..

[40] Finneran C.M., Zhang P. (2003). A Person–Artefact–Task (PAT) Model of Flow Antecedents in Computer-Mediated Environments. Int. J. Hum. Comput. Stud..

[41] Hirao K., Kobayashi R., Okishima K., Tomokuni Y. (2012). Flow Experience and Health-related Quality of Life in Community Dwelling Elderly Japanese. Nurs. Health Sci..

[42] Ley C., Krammer J., Lippert D., Barrio M.R. (2017). Exploring Flow in Sport and Exercise Therapy with War and Torture Survivors. Ment. Health Phys. Act..

[43] Gómez-Rico M., Santos-Vijande M.L., Molina-Collado A., Bilgihan A. (2023). Unlocking the Flow Experience in Apps: Fostering Long-term Adoption for Sustainable Healthcare Systems. Psychol. Mark..

[44] Bakker A.B., Oerlemans W., Demerouti E., Slot B.B., Ali D.K. (2011). Flow and Performance: A Study among Talented Dutch Soccer Players. Psychol. Sport Exerc..

[45] Petty R.E., Cacioppo J.T. (1986). The Elaboration Likelihood Model of Persuasion. Communication and Persuasion.

[46] Crowe E., Higgins E.T. (1997). Regulatory Focus and Strategic Inclinations: Promotion and Prevention in Decision-Making. Organ. Behav. Hum. Decis. Process..

[47] Tsai J.-M., Hung S.-W., Lin G.-T. (2022). Continued Usage of Smart Wearable Devices (SWDs): Cross-Level Analysis of Gamification and Network Externality. Electron. Mark..

[48] Jeong S.C., Choi B.-J. (2022). Moderating Effects of Consumers’ Personal Innovativeness on the Adoption and Purchase Intention of Wearable Devices. Sage Open.

[49] Kim H.-M., Cho I., Kim M. (2023). Gamification Aspects of Fitness Apps: Implications of MHealth for Physical Activities. Int. J. Hum. Comput. Interact..

[50] Stanley D.M., Cumming J., Standage M., Duda J.L. (2012). Images of Exercising: Exploring the Links between Exercise Imagery Use, Autonomous and Controlled Motivation to Exercise, and Exercise Intention and Behavior. Psychol. Sport Exerc..

[51] Zaichkowsky J.L. (1985). Measuring the Involvement Construct. J. Consum. Res..

[52] Hair J.F., Black W.C., Babin B.J., Anderson R.E., Tatham R.L. (2006). Multivariate Data Analysis.

[53] Fornell C., Larcker D.F. (1981). Evaluating Structural Equation Models with Unobservable Variables and Measurement Error. J. Mark. Res..

[54] Kline R.B. (2015). Principles and Practice of Structural Equation Modeling.

[55] Fan L., Liu X., Wang B., Wang L. (2017). Interactivity, Engagement, and Technology Dependence: Understanding Users’ Technology Utilisation Behaviour. Behav. Inf. Technol..

[56] Dvorak J.L. (2007). Moving Wearables into the Mainstream: Taming the Borg.

[57] Hong J.-C., Lin P.-H., Hsieh P.-C. (2017). The Effect of Consumer Innovativeness on Perceived Value and Continuance Intention to Use Smartwatch. Comput. Human Behav..

[58] Uzir M.U.H., Al Halbusi H., Lim R., Jerin I., Hamid A.B.A., Ramayah T., Haque A. (2021). Applied Artificial Intelligence and User Satisfaction: Smartwatch Usage for Healthcare in Bangladesh during COVID-19. Technol. Soc..

[59] Luo Y., Yang L., Ye Q., Liao Q. (2023). Effects of Customization and Personalization Affordances on Perceived Value and Continuance Intention of Smartwatch Use. Technol. Forecast. Soc. Chang..

[60] Molanorouzi K., Khoo S., Morris T. (2015). Motives for Adult Participation in Physical Activity: Type of Activity, Age, and Gender. BMC Public Health.

[61] Ingledew D.K., Markland D. (2008). The Role of Motives in Exercise Participation. Psychol. Health.

[62] Maltby J., Day L. (2001). The Relationship between Exercise Motives and Psychological Weil-Being. J. Psychol..

[63] Kim D., Kim A., Kim J., Ko Y.J. (2020). Symbiotic Relationship Between Sport Media Consumption and Spectatorship: The Role of Flow Experience and Hedonic Need Fulfillment. J. Glob. Sport Manag..

[64] Oh J., Kim D.H., Kim D. (2022). Exploring Experiential Patterns Depending on Time Lapses in Virtual Reality Spectatorship (VRS): The Role of Interruption in Reducing Satiation. Sustainability.

